# A comparison of diagnostic utility of new endoscopic scraper combined cell block method and conventional brush catheter for biliary tract cancer

**DOI:** 10.1002/deo2.331

**Published:** 2024-01-19

**Authors:** Noriyuki Kuniyoshi, Koji Yamada, Rota Osawa, Shuzo Nomura, Mariko Fujisawa, Kei Saito, Hiroo Imazu, Shinobu Masuda, Hirofumi Kogure

**Affiliations:** ^1^ Department of Gastroenterology and Hepatology Nihon University Itabashi Hospital Tokyo Japan; ^2^ Department of Gastroenterology Nihon University Hospital Tokyo Japan; ^3^ Department of Pathology Nihon University Itabashi Hospital Tokyo Japan

**Keywords:** biliary tract cancer, cell block, conventional brush catheter, malignant biliary stricture, trefle

## Abstract

**Background/Aims:**

The sensitivities of endoscopic *trans*‐papillary pathologic diagnosis of biliary tract cancer (BTC) are unsatisfactory. Recently, the diagnostic utility of the endoscopic scraper device, Trefle for biliary stricture has been reported. The Trefle can be guided to the target biliary stricture over the guidewire and is as easy to use as the conventional brush catheter (CBC). This study evaluated the efficacy and safety of Trefle‐assisted tissue acquisition combined cell block method and CBC cytology for biliary strictures due to BTCs.

**Methods:**

We retrospectively reviewed consecutive patients with biliary strictures in whom CBC cytology or Trefle‐assisted tissue acquisition under endoscopic retrograde cholangiopancreatography was performed for suspected BTCs from January 2015 to June 2022 at our institution.

**Results:**

173 patients (CBC group; *n* = 55, Trefle group; *n* = 118) were enrolled in this study. The sensitivity, specificity, and accuracy of CBC cytology for BTC were 68.3%/100%/76.4%. On the other hand, the sensitivity, specificity, and accuracy of Trefle‐assisted tissue acquisition for BTC were 93.7%/95.7%/94.1%, showing superior sensitivity (*p* < 0.001) and accuracy (*p* = 0.002) compared to that of CBC.

**Conclusions:**

Compared to CBC cytology, Trefle‐assisted tissue acquisition has superior diagnostic performance while maintaining procedural simplicity and is considered useful for diagnosing malignant biliary stricture.

## INTRODUCTION

Biliary tract cancer (BTC), including cholangiocarcinoma (CCA), gallbladder cancer (GBC), and ampullary tumors, is increasing in incidence.^1^ CCAs are divided into three subtypes depending on their anatomical site of origin: intrahepatic (iCCA), perihilar (pCCA), and distal (dCCA) CCA.^2^ BTCs have a very poor prognosis because it is difficult to diagnose at early stages and is often detected at advanced stages with unresectable or metastatic tumor.^3^, ^4^ Therefore, establishing a reliable diagnostic method at an early stage is desirable.

The pathological diagnosis of BTCs is commonly performed through bile aspiration cytology, biliary brush cytology, and forceps biopsy using endoscopic retrograde cholangiopancreatography (ERCP). However, their sensitivity to diagnose BTC is insufficient.^5^, ^6^, ^7^ Recently, peroral cholangioscopy (POCS) and POCS‐guided forceps biopsy (POCS‐FB) have been used to diagnose malignant biliary lesions. However, the specimens obtained by POCS‐FB are usually small, and pathological diagnosis is often difficult.^8^ Trefle is a new scraper device for biliary tract tissue collection that enables simultaneous cytological and histological diagnosis, and the diagnostic utility of this device for BTC has recently been reported.^9^, ^10^, ^11^, ^12^ As with a conventional brush catheter (CBC), Trefle can be guided to the target biliary stricture over the guidewire. Thus, this device can obtain tissues and cell samples with the same techniques as CBC and is superior in the procedural simplicity of sample collection. Although few studies compare the diagnostic utility of Trefle and CBC for BTC, their sensitivity is unsatisfactory.^11^, ^12^ Our institution utilizes the cell block method for processing specimens collected with Trefle to improve pathological diagnostic performance. In this study, we compared the diagnostic performance of Trefle‐assisted tissue acquisition combined cell block method and CBC cytology for biliary strictures due to BTCs and evaluated the usefulness of Trefle.

## METHODS

### Study population

The pathological diagnostic method for malignant biliary stricture suspected of BTC at our hospital was brush cytology before February 2017, but since then, we have performed Trefle‐assisted tissue acquisition. This study was a retrospective review of consecutive patients with biliary strictures in whom brush cytology or Trefle‐assisted tissue acquisition under ERCP was performed for suspected BTCs from January 2015 to June 2022 at Nihon University Itabashi Hospital, Tokyo, Japan. Pancreatic cancer and malignant biliary stricture due to lymph node metastasis were excluded from this analysis.

### Endoscopic procedure

After selective biliary cannulation with contrast or wire guidance, cholangiography was performed with a contrast medium to evaluate the biliary stricture. A 0.025‐inch angle‐tip guidewire (Visiglide2; Olympus and EndoSelector; Boston Scientific Japan) or a 0.035‐inch angle‐tip guidewire (RevoWave SeekMaster; Piolax Medical Devices, Inc.) was advanced through the biliary stricture, and then brush cytology or Trefle‐assisted tissue acquisition was performed over the guidewire before drainage catheter placement. For patients for whom endoscopic sphincterotomy (EST) was deemed necessary, it was done with a sphincterotome (KD‐V411M‐0725; Olympus Medical Systems) prior to specimen collection. Backward‐oblique viewing duodenoscope (JF260V, TJF 260V, and TJF‐Q290V; Olympus Medical Systems and ED‐580T; Fujifilm Medical Co.) was used in patients with normal anatomy, and single balloon enteroscope (SIF‐H290S; Olympus Medical Systems) or double balloon enteroscope (EI‐580BT; Fujifilm Medical Co.) in patients with the surgically altered anatomy.

### Trefle‐assisted tissue acquisition

**FIGURE 1:**
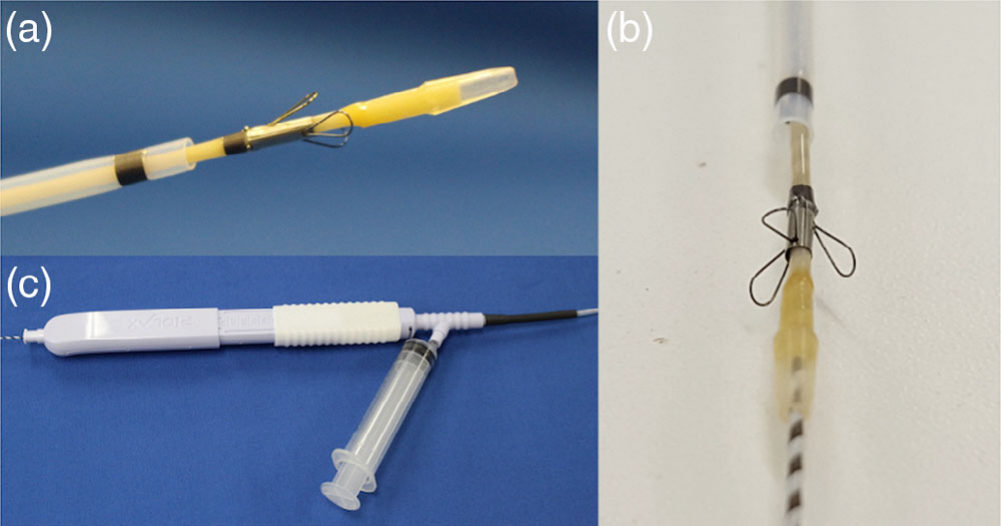
Appearance of Trefle. Trefle has three tiny metallic loops of 1.6 mm in diameter and is oriented at an angle of 120°. It also has a side port of the outer sheath from which tissue and cell samples with bile juice can be aspirated.

**FIGURE 2:**
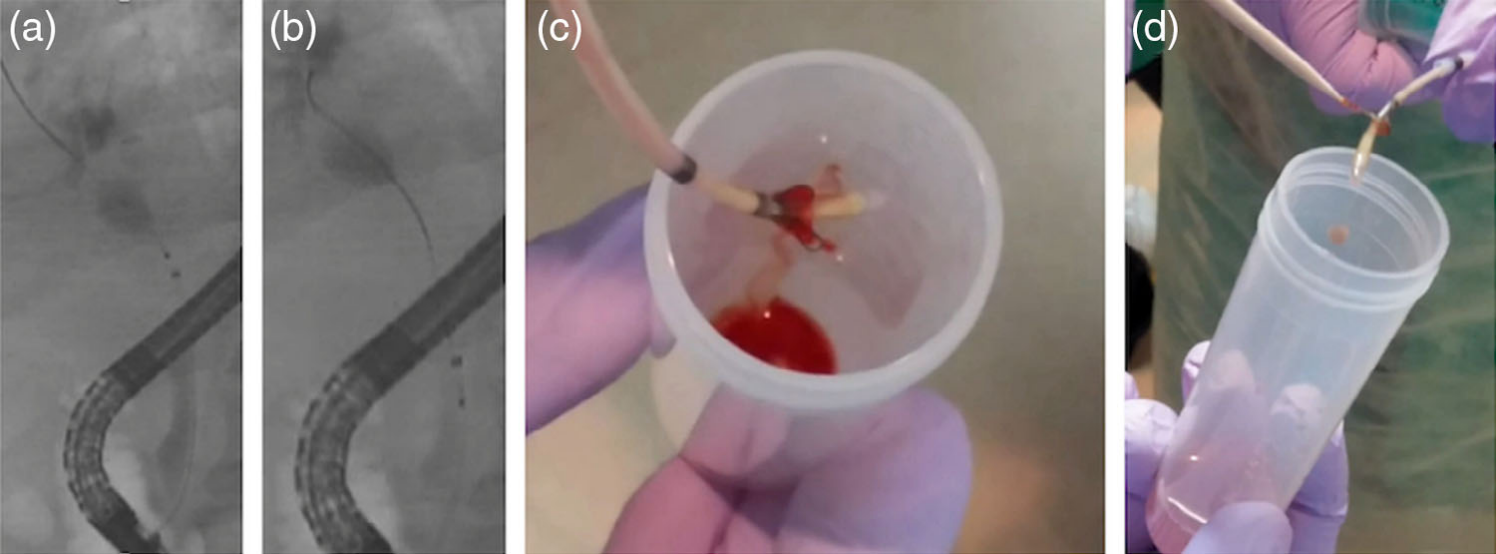
The method of Trefle‐assisted tissue acquisition. (a) Trefle was inserted into the biliary tract over the guidewire. Next, the loops of the device were opened and passed through the stricture under X‐ray fluoroscopy. (b) The loop is retracted when the device crosses the proximal end of the stricture. (c) All specimens including aspirated bile juice and tissues were transferred to a sterile tube (d) If tissue remains in the metaric loop, it is collected using a toothpick or similar tool.

The new endoscopic scraper, Trefle (PB7‐3L5S; Piolax Medical Devices, Inc.), having three tiny metallic loops of 1.6 mm in diameter and oriented at an angle of 120° (Figure 1), was inserted into the biliary tract over the guidewire. Next, the loops of the device were opened and passed through the stricture under X‐ray fluoroscopy. The loops are retracted when the device crosses the proximal end of the stricture. This procedure is repeated three times, and scraped tissues and/or cell samples with bile juice were obtained by aspiration through the side port of the outer sheath into a 10 mL syringe in the third procedure. All specimens, including aspirated bile juice and tissues, were transferred to a sterile tube (Figure 2).

### Specimen processing methods for histological and cytological diagnosis with Trefle

**FIGURE 3:**
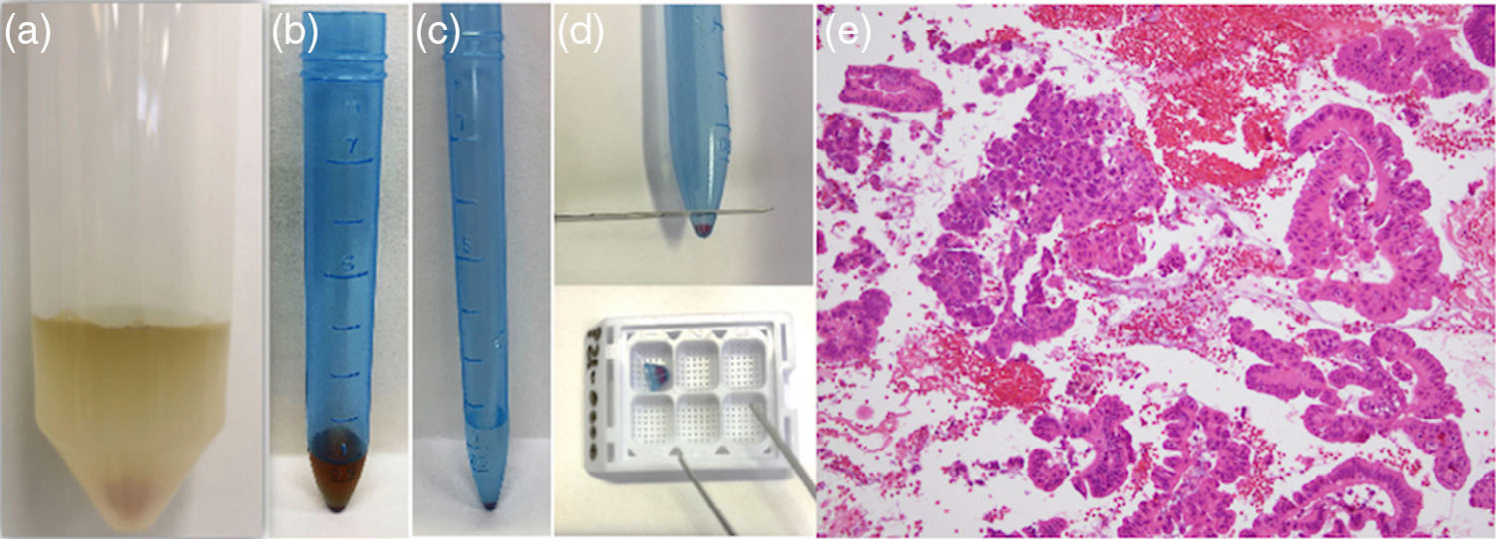
Specimen processing methods for histological and cytological diagnosis with Trefle. (a) Tissue specimens and cell samples were obtained by Trefle. (b) Approximately 70% of the liquid portion in addition to the tissue specimens large enough to be grasped with tweezers, was placed in a cotton swab tube. The remaining 30% of the liquid portion is used for cytology. (c) The deposit was obtained after centrifugation and fixed in formalin overnight. (d) A split section of sediment obtained by cutting a cotton swab tube with a scalpel is embedded in a cassette, dehydrated, and permeated with paraffin, yielding a cell block. (e) hematoxylin and eosin staining revealed adenocarcinoma in specimens obtained from the biliary stricture (H&E,×20).

Since submitted specimens are mixtures of bile, contrast media, and saline, the samples are processed promptly after confirming their properties. The specimen processing methods for histological and cytological diagnosis with Trefle are shown in Figure 3. First, approximately 70% of the liquid portion, in addition to the tissue specimens large enough to be grasped with tweezers, was placed in a cotton swab tube and centrifuged, and then the centrifuged deposit was fixed in formalin overnight. Next, a split section of sediment obtained by cutting a cotton swab tube with a scalpel is embedded in a cassette, dehydrated, and permeated with paraffin to make a paraffin block, yielding a cell block. The remaining 30% of the liquid portion is used for cytology specimen processing. Cytodiagnosis was performed according to Papanicolaou's classification. For histology, hematoxylin and eosin staining were performed, and immunostaining was added if necessary.

### The methods of specimen collection and pathological evaluation of CBC

We used a cytology brush (RX Cytology Brush; Boston Scientific) for brush cytology. First, the outer sheath was inserted into the proximal end of the stricture and brushed about five times through the stricture under X‐ray fluoroscopy. After brushing was completed, the brush was retracted into the outer sheath. Then, the brush tip was collected and cut in a saline spit. In addition, the specimen in the sheath was aspirated with a syringe and submitted as a cytology specimen with a cut brush.

### Diagnostic criteria

As an evaluation of pathological diagnosis, “suspicious for malignancy”/“malignancy” was defined as cancer‐positive in both cytology and histology. The final diagnosis was based on the pathological findings of the resection specimen in surgical cases and the clinical course, such as tumor growth and metastasis, by imaging in non‐surgical cases. In cases of benign biliary stricture, the final diagnosis was based on negative results of endoscopic cytohistology and the consensus of the patient's clinical course and multimodal imaging tests, including computed tomography, endosonography, and/or magnetic resonance imaging.

### Definition of the terms

In this study, “the procedure time” refers to the whole endoscopic procedure. Trainees were defined as endoscopists with less than ten years of endoscopic experience.

## RESULTS

### Patient's characteristics and baseline

The characteristics of patients with biliary disease are shown in Table 1. During the study period, 173 patients underwent CBC cytology or Trefle‐assisted tissue acquisition under ERCP for suspected BTCs. Of these, 55 patients were diagnosed with biliary stricture by CBC and 118 patients by Trefle. The median age was 75 years in both groups. There was a trend toward a higher proportion of males in the Trefle group than in the CBC group, but there was no significant difference. The distal biliary tract was the most common site of biliary tract stricture in both groups, followed by hilar and intrahepatic, but there was no significant difference between the two groups in the ratio of each. The median length of biliary stricture was also 15 mm in both groups, with no significant difference between the two groups. The final diagnosis of biliary stricture is also shown in Table 1. Of the 55 patients in the CBC group, 14 had benign biliary strictures, and 41 had malignant biliary strictures. On the other hand, of 118 patients in the Trefle group, 23 had benign biliary strictures, and 95 had malignant biliary strictures, showing no difference in the ratio of benign to malignant. The procedure‐related data are shown in Table 2. The success rate of the procedure was 100% for both CBC cytology (55/55) and Trefle‐assisted tissue acquisition (118/118). The Trefle group tended to have a shorter procedure time and a higher trainee performance rate than the CBC group. There was no significant difference in EST procedure rates between the two groups.

**TABLE 1:** Characteristics of patients with biliary disease.

	CBC (*n* = 55)	Trefle (*n* = 118)	*p*‐value
Median age, years (range)	75 (44–91)	75 (50–93)	0.99^*1^
Sex, male/female	32/23	85/33	0.08^*2^
Location(intrahepatic/perihilar/distal)			
Length of stricture, mm (range)	15(5–50)	15(5–40)	0.64^*1^
Malignant/Benign	41/14	95/23	0.43^*2^
Type of BTC			
iCCA	5	4	
pCCA	13	28	
dCCA	20	43	
GBC	2	19	
Ampullary tumors	1	1	
Type of benign disease			
Chronic pancreatitis	2	5	
IgG4‐related disease	5	3	
Benign biliary stricture	7	15	

Abbreviations: BTC, Biliary tract cancer; CBC, Conventional brush catheter; CCA, cholangiocarcinoma; dCCA, distal cholangiocarcinoma; GBC, gallbladder cancer; iCCA, intrahepatic cholangiocarcinoma; pCCA, perihilar cholangiocarcinoma.

^*1^ *p*‐Value: Mann–Whitney U test.

^*2^ *p*‐Value: Chi‐square test.

**TABLE 2:** The procedure‐related data.

	CBC (*n* = 55)	Trefle (*n* = 118)	*p*‐value
Success of the procedure (Yes/No)	55/0	118/0	1.00^*2^
Median procedure time, minutes (range)	71 (28–166)	41 (15–117)	<0.01^*1^
Trainee's procedure (Yes/No)	42/13	110/8	<0.01^*2^
EST (Yes/No)	15/40	40/78	0.48^*2^

Abbreviations: CBC, Conventional brush catheter; EST, Endoscopic sphincterotomy.

^*1^ *p*‐Value: Mann–Whitney U test.

^*2^ *p*‐Value: Chi‐square test.

### Diagnostic ability of brush cytology and Trefle‐assisted tissue acquisition for BTC

The diagnostic ability of CBC cytology and Trefle‐assisted tissue acquisition for BTC are shown in Table 3. The sensitivity, specificity, and accuracy for diagnosing malignant biliary stricture by CBC were 68.3%/100%/76.4%. On the other hand, the sensitivity, specificity, and accuracy of cytological diagnosis by Trefle was 91.6%/100%/93.2%, and the sensitivity, specificity, and accuracy of histological diagnosis by Trefle was 72.6%/95.7%/77.1%, and when both were combined, the sensitivity, specificity, and accuracy were 93.7%/95.7%/94.1%. Compared to CBC, Trefle showed superior sensitivity (*p* < 0.001) and accuracy (*p* = 0.002) for the diagnosis of BTCs.

**TABLE 3:** The diagnostic ability of conventional brush catheter (CBC) cytology and Trefle‐assisted tissue acquisition for biliary tract cancer.

		Sensitivity, %	Specificity, %	PPV, %	NPV, %	Accuracy, %
CBC cytology		68.3	100	100	51.9	76.4
Trefle	Cytology	91.6	100	100	74.2	93.2
	Histology	72.6	95.7	98.6	45.8	77.1
	Cytology + histology	93.7	95.7	98.9	78.6	94.1

Abbreviations: CBC, conventional brush catheter; NPV, negative predictive value; PPV, positive predictive value.

### Adverse events

Adverse events associated with both CBC cytology and Trefle‐assisted tissue acquisition are shown in Table 4. Post‐ERCP pancreatitis (PEP) occurred in 11 (20.0%) in the CBC group and 13 (11%) in the Trefle group. The incidence of PEP tended to be higher in both groups, but the difference between the two groups was not significant.

**TABLE 4:** Adverse events associated with both conventional brush catheter cytology and Trefle‐assisted tissue acquisition.

	CBC (*n* = 55)	Trefle (*n* = 118)	*p*‐value
PEP	11 (20.0%)	13 (11.0%)	0.99^*^
Severe PEP	3 (5.5%)	5 (4.2%)	
Bleeding	1 (1.8%)	0 (0%)	0.07^*^

Abbreviations: CBC, conventional brush catheter; PEP, Post‐ERCP pancreatitis.

* *p*‐Value: Chi‐square test.

The PEP was considered severe in three (5.5%) patients in the CBC group and five (4.2%) in the Trefle group. One case of post‐EST bleeding was observed in the CBC group but improved with endoscopic therapy. Other adverse events or subsequent deaths were not observed.

## DISCUSSION

Pathology is necessary for cases of operable BTCs and cases of BTCs prior to induction of chemotherapy. Pathological diagnostic methods for BTCs include endoscopic trans‐papillary bile cytology, brush cytology, and forceps biopsy. However, the sensitivity of bile cytology for malignant biliary stricture is reported to be as low as 6%–32%.^6^ The sensitivity and specificity of brush cytology and biliary forceps biopsy for the diagnosis of BTC have been reported to be 44%/99% and 48%/99%, respectively, and the sensitivity and specificity of their combined evaluation are 59%/100%, but these results are also not sufficient.^7^ In recent years, POCS‐guided forceps biopsy has also been a common method of tissue acquisition in biliary stricture. Many studies have reported that POCS‐guided targeted biopsy has a higher sensitivity than a trans‐papillary forceps biopsy and brushing cytology.^13^ However, the specimens obtained by POCS‐guided biopsy are usually small, and pathological diagnosis is often difficult. Endoscopic ultrasound‐guided fine needle aspiration (EUS‐FNA) has also been used to diagnose BTC, and its diagnostic accuracy has been reported to be higher than that of trans‐papillary forceps biopsy and/or cytology (94% vs. 53%).^14^ EUS‐FNA for distal CCA can be performed relatively safely as in pancreatic cancer, but in the case of hilar CCA, the trans‐papillary pathological method is recommended due to the risk of peritoneal dissemination.^15^, ^16^ For these reasons, pathological diagnostic methods in BTC remain a challenge.

Trefle was designed to easily access biliary strictures and obtain both tissue and cell samples for histology and cytology. Sakuma et al. reported that Trefle could yield larger tissue volume and better diagnostic performance for BTC than conventional biliary forceps biopsy　(cancer detectability; Trefle vs. forceps biopsy = 64.7% vs. 51.3%).^9^ Kato et al. evaluated the diagnostic performance of Trefle‐assisted tissue acquisition and POCS‐guided forceps biopsy based on pathological evaluation for extrahepatic CCA (ECC) and reported the diagnostic ability of Trefle‐assisted tissue acquisition for ECC is similar to that of POCS‐guided tissue acquisition (accuracy; POCS vs. Trefle = 90.0% vs. 85.7%).^10^ These results indicate that Trefle‐assisted tissue acquisition is superior to conventional biopsy forceps for biliary biopsy in the diagnosis of BTC and may be comparable to POCS‐guided forceps biopsy. In clinical practice, transpapillary forceps biopsy remains the gold standard for tissue collection during ERCP. However, the free‐hand transpapillary insertion of forceps and penetration of the stricture requires skill. On the other hand, Trefle can be inserted into the target stricture over the guidewire like a CBC. Therefore, Trefle‐assisted tissue acquisition may be a safer and superior method than transpapillary forceps biopsy to diagnose BTC. Trefle may be used as the first line in the pathological diagnosis of BTC.

Trefle is comparable to brush cytology in terms of technique and difficulty, and there are few direct comparison studies between Trefle and CBC based on pathological evaluation for BTC.^11^, ^12^ However, their sensitivity of Trefle for BTC is not satisfactory. We used the cell block method to process specimens collected with Trefle to improve pathological diagnostic performance. Therefore, in this study, we compared the diagnostic performance of Trefle combined with our cell block method to CBC cytology for BTC and to the result of a previous report evaluating the utility of Trefle.

In our study, the diagnostic ability of Trefle for BTC was superior to that of CBC cytology and superior to the result of a previous report on the performance of Trefle.^9^, ^10^, ^11^, ^12^ Compared to the CBC group, procedure times were no longer in the Trefle group, despite the higher trainee performance rate. Nevertheless, the Trefle group had the better diagnostic ability for BTC results, suggesting that the Trefle may be a safe and efficient device for tissue acquisition of biliary strictures. PEP tended to be higher in both groups, although the differences were insignificant. The incidence of PEP in overall ERCP at our institution is about 3%. It should be noted that the incidence of PEP tends to be higher in the diagnostic and/or therapeutic ERCP for BTC.

Identifying tissue clumps in Trefle specimen processing is difficult because of the opacity of the surrounding fluid. In addition, tissue may remain in the fluid component. Therefore, a simpler and more efficient processing method is needed in the processing of Trefle‐assisted tissue acquisition. Kato et al. reported the usefulness of the cell block method with the Trefle,^12^, ^17^ and we also utilize the cell block method for processing specimens collected with Trefle. Using approximately 70% of the liquid portions with the tissue sample collected with Trefle for cell block preparation increased the proportion of specimens used as histological tests and decreased tissue fragmentation in the HE specimens. In the present study, the histological diagnostic ability of Trefle for BTC was superior to those reported previously.^10^, ^11^, ^12^ Two cases could not be diagnosed by cytology but could be only diagnosed by histology. This may be partly due to the ingenuity of the specimen processing method at our institution. On the other hand, the cytological diagnostic accuracy did not decline, although the proportion of specimens used for cytology was reduced. Although the diagnostic results of the cytology of Trefle were good in this study, diagnosis by cytology is inevitably dependent on the skill of the cytologist, which may lead to lower results when the same method is verified at other institutions. Therefore, we think it was effective that histological samples obtained by Trefle were also treated using the cell block method.

In recent years, the development of molecular‐targeted drugs and immune checkpoint inhibitors has increased the demand for screening for genetic alterations in BTC.^18^, ^19^ Therefore, not only cytological diagnosis but also the yield of sufficient tissue samples is becoming increasingly important. In addition, immunostaining tests can be performed on tissue collected by Trefle if sufficient quantities are available. In the present study, seven cases (5.9%; 7/118) underwent additional immunostaining tests and were ultimately diagnosed with BTC. The feasibility of immunostaining tests in these cases may be due to the cell block method, which decreased tissue fragmentation and increased the proportion of specimens. For these reasons, we believe that our specimen processing methods can improve the diagnostic ability of Trefle for BTC and should continue to be utilized. The actual usefulness of this method for cancer multi‐gene panel testing has not yet been examined but will need to be studied in the future.

There are several limitations to this study. First, this was a single‐center retrospective study without a comparative cohort. Second, because Trefle allows both cytology and histology, it would be desirable to compare the results of Trefle‐assisted tissue acquisition for BTC to that of the combination of CBC cytology and histology by biliary forceps biopsy, but this has not been possible. Therefore, multicenter, prospective, randomized comparative studies with such combined methods are needed to clarify the incremental utility of Trefle on pathological diagnosis of BTC. Third, the instructor differed between the period of Trefle use and CBC use, which may have affected both groups’ procedure time and trainee performance rate. However, the fact that the diagnostic performance of Trefle‐assisted tissue acquisition was better than CBC cytology for BTC despite more trainees performing the procedure in the Trefle group than in the CBC group strongly suggests the superiority of Trefle.

In conclusion, the diagnostic ability of Trefle‐assisted tissue acquisition for BTC was superior to that of CBC cytology. Trefle was considered an effective device that can be performed easily and safely in diagnosing BTC. This study also suggested that specimen processing combined with the cell block method of the sample obtained by Trefle may effectively improve diagnostic performance. We believe that sample acquisition under ERC with Trefle is useful for diagnosing malignant biliary stricture and should be used aggressively.

## CONFLICT OF INTEREST STATEMENT

None.
